# Electrocardiographic Changes Following Pediatric Adenotonsillectomy for Obstructive Sleep-Disordered Breathing: A Pilot Prospective Study

**DOI:** 10.3390/pediatric18050128

**Published:** 2026-09-27

**Authors:** Francisco Alves de Sousa, Mariline Santos, Marta Rios, Sofia Carvalho, Simão Bessa, Manuel Magalhães

**Affiliations:** 1 Otorhinolaryngology and Head & Neck Surgery Department, Unidade Local de Saúde de Santo António, 4099-001 Porto, Portugal; 2 Instituto de Ciências Biomédicas Abel Salazar, Universidade do Porto, 4099-002 Porto, Portugal; 3 Pediatrics Department, Unidade Local de Saúde de Santo António, 4099-001 Porto, Portugal; 4 Otorhinolaryngology and Head & Neck Surgery Department, Unidade Local de Saúde de Tâmega e Sousa, 4564-007 Penafiel, Portugal

**Keywords:** pediatric obstructive sleep-disordered breathing, children, adenotonsillectomy, electrocardiogram, P-wave, QRS amplitude

## Abstract

**Background/Objectives**: Adenotonsillar hypertrophy (ATH) is the primary driver of pediatric obstructive sleep-disordered breathing (OSDB), potentially leading to cardiac remodeling. This study evaluated the impact of adenotonsillectomy (AT) on electrocardiographic (ECG) parameters and correlated these changes with sleep metrics. **Methods**: This prospective study evaluated ECG parameters (P-wave and QRS morphology) in children (>3 years) with symptomatic ATH before and three months after AT. A subset underwent sleep analysis via Cardiopulmonary Coupling (CPC) at both time points. Pre- and postoperative data were compared using paired *t*-tests and Linear Mixed Models (LMMs). The link between ECG and sleep modifications was also analyzed using Pearson and Spearman correlations of their respective variations (Δ). **Results**: Significant reductions were observed in the maximum RR interval (*p* = 0.027). Mean P-wave duration significantly shortened (84.40 ± 6.51 ms to 81.20 ± 3.32 ms; *p* = 0.043) and mean amplitude increased (0.076 ± 0.008 mV to 0.080 ± 0.004 mV; *p* < 0.001). Mean, minimum, and maximum QRS amplitudes increased significantly (*p* ≤ 0.018). Exploratory LMM confirmed that these modifications remained significant after adjusting for age and sex. Furthermore, post hoc analyses revealed that Δ QRS maximum amplitude exhibited negative correlations that survived false discovery rate (FDR) adjustment with Δ AHI 4% max (r = −0.727, *p* = 0.045), Δ ODI 3% max (r = −0.765, *p* = 0.045), Δ ODI 4% mean (r = −0.819; *p* = 0.033), and Δ maximal hypoxic burden (r = −0.730; *p* = 0.045). **Conclusions**: AT may alter ECG parameters. QRS amplitude increments correlated well with a decrease in nocturnal respiratory events. These findings may represent preliminary ECG changes associated with postoperative respiratory improvement. Larger controlled studies are needed to determine whether these ECG parameters can serve as markers of post-operative cardiopulmonary recovery.

## 1. Introduction

Adenotonsillar hypertrophy (ATH) is one of the most common disorders in childhood and the primary cause of airway obstruction and obstructive sleep-disordered breathing (OSDB) [1,2,3]. Chronic upper airway obstruction leads to a cascade of physiological stressors, including recurrent hypoxemia, hypercarbia, and exaggerated negative intrathoracic pressure [4,5,6]. These mediators induce pulmonary vasoconstriction and increased sympathetic activity, which may result in right ventricular dysfunction, pulmonary hypertension, and atrial enlargement [5,7,8,9,10,11]. These parameters often normalize following adenotonsillectomy (AT), indicating that early cardiac remodeling in children is reversible [12,13,14,15,16,17].

In adults, OSDB is a well-established risk factor for atrial tachyarrhythmias, particularly atrial fibrillation, due to intra-atrial and inter-atrial conduction delays [18,19,20]. These delays are reflected on a standard electrocardiogram (ECG) as increased P-wave duration and P-wave dispersion (PWD) [18,20,21].

In children, while the cardiac implications of OSDB are increasingly recognized, most research focuses on echocardiographic markers like pulmonary artery pressure or myocardial performance indices [13,22,23,24]. Nevertheless, recent literature suggests that P-wave markers are significantly increased in symptomatic ATH compared to healthy controls [12,25,26]. However, these studies utilized traditional 12-lead ECG monitoring, which is often cumbersome for longitudinal pediatric follow-up.

In this study, we investigate the effect of surgical treatment of pediatric ATH in cardiac conduction by utilizing a single-lead ECG analysis. By focusing on the temporal and morphological changes in the P-wave and QRS, we aim to determine if these specific conduction parameters can serve as preliminary indicators for nocturnal recovery. We hypothesize that the resolution of airway obstruction following AT may lead to quantifiable alterations and that these electrical variations (deltas) could correlate with the improvement in nocturnal respiratory metrics, such as the Apnea–Hypopnea Index (AHI).

## 2. Materials and Methods

### 2.1. Study Design and Population

This prospective investigation evaluated children over three years of age scheduled for elective outpatient AT secondary to symptomatic ATH. Clinical indications for surgery were established through a comprehensive clinical history reported by caregivers (including habitual snoring, witnessed apneas, and persistent mouth breathing), which were then verified through physical examination and standardized grading of tonsillar (Brodsky ≥ 2) and adenoid tissue (Clemens and McMurray ≥ 2). Patients with a body mass index (BMI) > 95th percentile, identified craniofacial syndromes, neuromuscular diseases, or pre-existing cardiovascular comorbidities were excluded. All participants underwent standard extracapsular AT using identical cold dissection, and no major perioperative complications were recorded. Detailed exclusion criteria and the comprehensive study workflow are illustrated in Figure 1. To ensure post-surgical cardiovascular stabilization, follow-up assessments were conducted three months postoperatively and compared against baseline preoperative measurements. Given the prospective and pilot nature of this investigation, a pragmatic sample size was utilized, consisting of all consecutive patients who met the inclusion criteria during the designated study window. The reporting of this study conforms to the Strengthening the Reporting of Observational Studies in Epidemiology (STROBE) guidelines for cohort studies.

### 2.2. Electrocardiographic Analysis

Electrocardiogram recordings were obtained using the KardiaMobile^®^ handheld single-lead monitor (AliveCor, Inc., Mountain View, CA, USA), an FDA-approved device. Following manufacturer protocols, participants rested in a quiet environment while maintaining bilateral contact with the electrodes to establish a circuit between the left and right hands. Data were transmitted via a Bluetooth connection to a proprietary application (Kardia, Version 5.61.1) hosted on a tablet computer (iPad; Apple Inc., Cupertino, CA, USA), enabling the acquisition of a 30-s Lead I-equivalent rhythm strip. This frontal plane tracing was utilized for morphometric analysis.

Manual morphometric analysis was conducted on the high-resolution rhythm strips by visually inspecting the standardized PDF grid. Measurements were calibrated to the device’s recording standards of 25 mm/s (horizontal) and 10 mm/mV (vertical), where each 1 mm small square corresponded to 0.04 s and 0.1 mV, respectively. To obtain the reported sub-millimeter measurement precision, digital magnification (400% calibrated zoom) of the exported high-resolution PDF tracings was utilized, allowing visual resolution down to 0.01 mV and 2 ms. For each 30-s tracing, an average of five consecutive cardiac cycles were sampled from stable segments to determine mean, minimum, and maximum values for most parameters. Heart rate was derived from the average of consecutive R-R intervals. P-wave and QRS durations were measured from the initial deflection of the waveform to its return to the isoelectric baseline. Amplitudes were quantified as the peak vertical displacement from the isoelectric line. To account for physiological variability and ensure precision, the maximum (Pmax) and minimum (Pmin) durations were recorded across the entire tracing. All measurements were performed by a single investigator who was blinded to the clinical timepoint (preoperative vs. postoperative) to ensure intra-rater consistency and minimize detection bias. To evaluate intra-observer reliability, a random subsample of 10 tracings was measured twice, three months apart. The Intraclass Correlation Coefficient (ICC, two-way mixed effects, absolute agreement, single rater) demonstrated high reliability. Specifically, when grouped by metric type, the ICC point estimates ranged from 0.93 to 0.97 (95% CIs ranging from 0.88 to 0.98) for duration variables (P-wave and QRS duration) and from 0.92 to 0.96 (95% CIs ranging from 0.85 to 0.98) for amplitude variables. However, while this high relative reliability confirms consistency, it does not independently demonstrate that absolute mean changes as small as 0.004 mV entirely exceed the standard error of measurement (SEM); thus, these fine metrics must be interpreted with inherent technical caution.

### 2.3. Sleep Cardiorespiratory Assessment

A subgroup of 11 children underwent multi-night domiciliary sleep monitoring using the SleepImage^®^ system (MyCardio LLC., Denver, CO, USA), an FDA-cleared Software-as-a-Medical-Device, clinically validated in pediatric populations aged 2 years and older [27]. Participation in this subgroup was determined strictly by device availability during the patient’s testing window and parental consent to perform multi-night recordings. This system utilizes Cardiopulmonary Coupling (CPC) technology to evaluate sleep quality and respiratory effort through the analysis of a single-lead photoplethysmogram (PPG) signal. By quantifying the coherent interaction between Heart Rate Variability (HRV) and Pulse-Derived Respiration (PDR), the CPC algorithm categorizes sleep into Stable (High-Frequency Coupling; parasympathetic dominance) and Unstable (Low-Frequency Coupling; sympathetic dominance) states [27,28].

To mitigate the “first-night effect” and account for the inherent night-to-night variability characteristic of pediatric sleep architecture, a multi-night domiciliary recording protocol was implemented. Inclusion in the final analysis required a minimum of 180 min of continuous sleep recording with high signal integrity, a threshold established to ensure a physiologically representative assessment of sleep [5]. Inadequate nights failing to meet this threshold were excluded from the analysis. Given the longitudinal nature of the multi-night data, both mean and peak severity (worst-case) values were recorded to facilitate a more comprehensive characterization of the subjects’ sleep profiles. The “nightly mean” was defined as the mathematical average of a given metric across all valid recording nights for a participant, while the “peak” value represented the single worst recorded night to capture maximal physiological severity. Key output metrics included the Oxygen Desaturation Index (ODI) and Apnea–Hypopnea Index (AHI) both at 3% and 4% thresholds, hypoxic burden (cumulative area of oxygen desaturation per hour of sleep (%min/h)), oxygen saturation nadir, Respiratory Disturbance Index (RDI), and respiratory event duration (in seconds).

### 2.4. Ethics

Adhering to the ethical standards established by the Declaration of Helsinki, this research was carried out following the formal approval of the institutional Ethics Committee of Instituto de Ciências Biomédicas Abel Salazar (ICBAS)/Unidade Local de Saúde de Santo António (ULSSA) (protocol code: 2021.234 (186-DEFI/194CE)). To uphold the principles of voluntary participation and confidentiality, written informed consent was secured from the legal guardians or caregivers of each child prior to the start of any data acquisition.

### 2.5. Statistical Analysis

Data were analyzed using using IBM SPSS Statistics, Version 29.0.0.0 (IBM Corp., Armonk, NY, USA). Paired-samples *t*-tests were employed to evaluate primary physiological changes between pre- and postoperative states. Normality assumptions for the paired differences were assessed using the Shapiro–Wilk test. For variables exhibiting asymmetric distributions, particularly within the small sleep-monitored subgroup, non-parametric Wilcoxon signed-rank tests were performed as sensitivity analyses, confirming the direction and significance of the primary findings. To account for potential confounding factors and examine the stability of the surgical impact, exploratory Linear Mixed Models (LMMs) were employed for selected ECG variables demonstrating significant or borderline-significant (*p* < 0.06) bivariate changes. Within these models, Time (pre- vs. postoperative) was treated as the primary factor, while Sex and Age were introduced as the fixed factor and covariate, respectively, to evaluate their main effects and potential interactions with the procedural outcome. The models included a random intercept for each participant to account for repeated measures, utilized a variance-component covariance structure, and applied Restricted Maximum Likelihood (REML) estimation. Degrees of freedom were calculated using Satterthwaite approximations. To assess the relationship between cardiac dynamics and sleep architecture, bivariate Pearson and Spearman correlations were performed on the delta (Δ) variations in ECG metrics and sleep parameters. Because the correlation analyses within the sleep-monitored subgroup (n = 11) were initially exploratory and intended for hypothesis generation, the displayed *p*-values were not adjusted for multiple comparisons; consequently, such findings should be interpreted with caution. To overcome this limitation, we conducted a post hoc exploratory analysis defining a specific family of tests based on our primary LMM outcomes: the relationship between Δ QRS amplitude max and the assessed sleep metrics was designated as the target correlation family, and *p*-values were adjusted using the Benjamini–Hochberg False Discovery Rate (FDR) procedure. Furthermore, the numerous paired ECG comparisons themselves represent a family of multiple tests; therefore, their nominal *p*-values should be interpreted alongside effect estimates and confidence intervals as exploratory findings. Statistical significance was defined as *p* < 0.05 for all analyses.

## 3. Results

### 3.1. Participant Flow and Characteristics

#### 3.1.1. Sample Selection

In total, 37 patients were identified as meeting the inclusion criteria, of whom 31 attended the baseline preoperative assessment. Following the loss of six patients during the three-month follow-up period, the final analytical cohort consisted of 25 participants who completed both preoperative and postoperative electrocardiographic evaluations (Figure 1). Participants lost to follow-up did not differ significantly from the final cohort in terms of baseline age, sex, or OSDB clinical severity (*p* > 0.05).

#### 3.1.2. Age, Sex, and Clinical Baseline

The mean age of participants at surgery was 6.68 ± 2.38 years, with a minimum of 3 years and a maximum of 12 years. There were 14 males (56%) and 11 females (44%). No statistically significant difference in age at the time of surgery between males and females was noted (*p* = 0.694). The cohort predominantly exhibited significant ATH, with 88% presenting Brodsky grade III or IV tonsils. The mean BMI percentile of the included participants was 58.4 ± 14.2%, with no subjects classified as obese.

### 3.2. Primary Objective: Electrocardiographic Parameter Changes

Analysis of the study cohort revealed significant modifications in electrocardiographic parameters following surgical intervention, as displayed in Table 1.

#### 3.2.1. Bivariate Analysis: Paired-Samples *t*-Test

##### Heart Rate and RR Interval 

Resting heart rate showed a slight increase (87.84 ± 10.89 bpm to 90.52 ± 10.07 bpm), but this variation was not statistically significant (*p* = 0.250). However, a significant reduction was observed in the maximum RR interval, which decreased from 800.80 ± 117.82 ms to 737.20 ± 96.50 ms (*p* = 0.027).

##### Atrial Conduction Time Narrowing 

The mean P-wave duration narrowed significantly from 84.40 ± 6.51 ms preoperatively to 81.20 ± 3.32 ms postoperatively (*p* = 0.043).

##### Atrial Conduction Amplitude Increase 

Mean P-wave amplitude demonstrated a significant elevation, rising from 0.076 ± 0.008 mV to 0.080 ± 0.004 mV (*p* < 0.001). This was further supported by a significant increase in minimum P-wave amplitude (0.047 mV to 0.051 mV, *p* = 0.009).

##### Ventricular Substrate Modifications 

Although no significant change was found in QRS duration (*p* = 0.356), there were significant increases across all QRS amplitude metrics, including mean (*p* = 0.003), minimum (*p* = 0.018), and maximum (*p* = 0.016) amplitudes.

#### 3.2.2. Multivariate Analysis: Linear Mixed Models

To further investigate how demographic factors influenced surgical outcomes, Linear Mixed Models (LMMs) were employed for parameters demonstrating statistically significant or borderline-significant shifts in the bivariate analysis. Sex was taken as a fixed factor and age as a covariate. As detailed in Table 2, the adjusted effect of time (surgery) remained significant for the majority of modeled parameters conditional on these covariates.

Specifically, for the modeled markers of atrial conduction, demographic variables showed no significant influence on RR interval max (Sex: F[1, 21] = 0.41, *p* = 0.531; Age: F[1, 21] = 1.47, *p* = 0.238; interactions: *p* ≥ 0.260) and mean P-wave duration (Sex: F[1, 21] = 0.25, *p* = 0.620; Age: F[1, 21] = 1.20, *p* = 0.282; interactions: *p* ≥ 0.218). Similarly, age and sex did not significantly modulate changes in atrial conduction amplitude, namely mean P-wave amplitude (Sex: F[1, 21] = 1.56, *p* = 0.220; Age: F[1, 21] = 0.61, *p* = 0.439; interactions: *p* ≥ 0.228) and minimum P-wave amplitude (Sex: F[1, 21] = 0.04, *p* = 0.840; Age: F[1, 21] = 0.01, *p* = 0.911; interactions: *p* ≥ 0.754).

Ventricular depolarization markers demonstrated a similar pattern where the adjusted effect of time remained significant conditional on covariates regarding mean QRS amplitude (Sex: F[1, 21] = 0.10, *p* = 0.751; Age: F[1, 21] = 0.29, *p* = 0.592; interaction: *p* ≥ 0.597), minimum QRS amplitude (Sex: F[1, 21] = 0.01, *p* = 0.924; Age: F[1, 21] = 0.01, *p* = 0.922; interactions: *p* ≥ 0.199), and maximum QRS amplitudes (Sex: F[1, 21] = 3.96, *p* = 0.060; Age: F[1, 21] = 0.12, *p* = 0.73; interactions: *p* ≥ 0.404).

### 3.3. Secondary Objective: ECG and Sleep Metrics’ Correlation Analysis

#### 3.3.1. Describing Sleep Metrics

A subset of n = 11 patients performed pre- and postoperative sleep tests. The sleep-monitored and non-monitored (n = 14) groups were comparable regarding age, sex, baseline clinical metrics, and all postoperative ECG metrics at the three-month follow-up (*p* > 0.05 for all variables). Within the monitored subset, the mean number of nights recorded per subject was 3.47 ± 1.73 preoperatively and 2.9 ± 1.70 postoperatively (*p* = 0.167). Similarly, the mean recording duration did not significantly vary between the preoperative (361.98 ± 146.10 min) and postoperative (338.75 ± 133.59 min) assessments (*p* = 0.564). Table 3 displays the main sleep metrics obtained for this group.

#### 3.3.2. Correlation Analysis of Deltas (Δ)

The correlation matrix of cardiorespiratory improvements (Figure 2) indicated that the Δ QRS amplitude mean was significantly and negatively associated with the Δ ODI 4% mean (r = −0.616, *p* = 0.044). Furthermore, the Δ QRS amplitude max demonstrated several negative correlations, specifically with the Δ AHI 4% max (r = −0.727, *p* = 0.011), Δ ODI 3% max (r = −0.765, *p* = 0.006), the Δ ODI 4% mean (r = −0.819, *p* = 0.002), the Δ ODI 4% max (r = −0.701, *p* = 0.016), and the Δ Hypoxic Burden max (r = −0.730, *p* = 0.011). A moderate correlation was observed between Δ mean P-wave duration and Δ obstructive event duration (r = 0.512); however, this relationship did not reach statistical significance (*p* = 0.107). No other statistically significant correlations were observed in the unadjusted matrix (*p* > 0.05). It is critical to differentiate between the broad, unadjusted correlation matrix (Figure 2)—which should be interpreted strictly as an exploratory, hypothesis-generating analysis—and the targeted post hoc FDR analysis (Table 4).

Based on the strongest LMM outcomes, we defined a specific post hoc family comprising the correlations between Δ QRS amplitude max and all assessed sleep metrics. Within this complete defined family (Table 4), four associations successfully survived FDR correction, specifically with the Δ ODI 4% mean, the Δ ODI 3% max, the Δ AHI 4% max, and the Δ Hypoxic Burden max.

## 4. Discussion

Increasing evidence points to the multisystemic impact of pediatric OSDB, particularly regarding autonomic dysregulation and cardiovascular sequelae [29]. Our preliminary pilot results suggest that surgical resolution of ATH may induce electrocardiographic alterations in the pediatric population. Our findings establish two primary insights: first, that atrial and ventricular conduction may change within three months of intervention, and second, that these electrical modifications may be linked to the reduction in hypoxic burden and respiratory events during sleep. Also, by utilizing portable single-lead ECG and CPC sleep metrics, this study describes a potentially simple, non-invasive method for cardiovascular analysis in children with OSDB.

### 4.1. Atrial Conduction and Electromechanical Changes

One primary finding of this investigation is the significant narrowing of mean P-wave duration (84.40 ms to 81.20 ms, *p* = 0.043) following AT. This aligns with the landmark evidence reported by Kocabas et al., who identified that preoperative atrial conduction delays in children with ATH resolve effectively post-surgery [12]. This electrophysiological change is further substantiated by Tissue Doppler Imaging (TDI) studies showing that atrial electromechanical delays—prolonged in symptomatic children—normalize postoperatively across both intra-atrial and inter-atrial segments [12].

Pathophysiologically, chronic upper airway obstruction generates exaggerated negative intrathoracic pressure and recurrent alveolar hypoxia, precipitating pulmonary vasoconstriction and increased right ventricular (RV) afterload, while increasing sympathetic activity [4,6,8,9,15,30]. The resulting right atrial (RA) stretch facilitates conduction slowing and heterogeneity through the activation of stretch-activated ion channels and geometric changes in atrial architecture [31,32]. In our study, the observed significant reduction in P-wave duration indicates that relieving this mechanical “nocturnal load” may hypothetically facilitate more homogeneous atrial activation.

Furthermore, we observed a significant increase in mean and minimum P-wave amplitude. While increased amplitude is traditionally associated with “P-pulmonale” in chronic obstructive states [33], the postoperative voltage increase in our cohort more likely reflects possible electrophysiological changes. In the pediatric model, short-term stretch exposure has presumably not yet induced irreversible fibrosis; thus, removing the obstructive stimulus could hypothetically permit reverse remodeling, which is often incomplete in adult OSA populations [2,13,24,31,32,34,35].

### 4.2. Ventricular Dynamics and Autonomic Stabilization

While mean heart rate remained stable, we identified a significant reduction in the maximum RR interval (800.80 ms to 737.20 ms, *p* = 0.027). In fact, this suggests a specific attenuation of peak periodicities rather than a global chronotropic shift. While it could also be hypothesized to reflect thewithdrawal of extreme phasic parasympathetic influences (the “vagal peaks”) associated with obstructive events [8], the daytime acquisition of these recordings precludes such direct causal attribution. In fact, diurnal carry-over effects of nocturnal autonomic stabilization have been reported by previous works [29,36], but this reduction in daytime RR interval may also be explained by variations in recording conditions, subtle changes in daytime autonomic tone, or statistical regression to the mean.

While adult studies have associated the severity of OSDB with diminished QRS voltage, longitudinal data tracking QRS morphometry following treatment remain scarce [37], particularly in pediatric populations. Addressing this notable gap in the literature, our analysis identified a significant postoperative increase in QRS amplitude across all metrics. At present, these changes are best described as morphometric findings observed following surgery. Because this is an uncontrolled before-and-after study, changes in QRS amplitude cannot presently be regarded as evidence of improved myocardial performance or “ventricular electrical recovery.” While echocardiographic research demonstrates that Myocardial Performance Indices (MPIs) normalize within three months of AT [2,9,13,24], our study does not include concurrent echocardiographic, structural, or functional cardiac measurements. Also, the resolution of hypoxic pulmonary vasoconstriction has been reported to reduce right ventricular afterload, which may enhance depolarization efficiency and electrical stability across the ventricular substrate [9,13,24], but our single-lead ECG data cannot definitively confirm if these specific mechanisms drive such morphometric changes. Therefore, these alterations must be interpreted with strict caution, as QRS amplitude in a single lead is highly sensitive to alternative non-cardiac explanations, including normal somatic growth, postoperative changes in body composition, variable electrode contact, hand position, skin impedance, recording conditions, and regression to the mean.

### 4.3. ECG Morphometry as an Exploratory Indicator for Respiratory Recovery

The exploratory associations between our ECG findings and respiratory data are best illustrated by the significant inverse correlations between Δ QRS maximal amplitude and several indices of respiratory severity, including AHI 4% max, ODI (3% and 4%) max, and maximal hypoxic burden. These associations raise the question if the magnitude of ventricular QRS amplitude increase may mirror the degree of nocturnal airway obstruction relief. These results partially diverge from those of Isaiah et al., who analyzed CHAT trial data [38] and concluded that postoperative heart rate variability (HRV) improvements were not causally linked to changes in AHI or oxygen desaturation specifically [39]. In contrast, our data suggest a relationship between ventricular depolarization and the mitigation of hypoxic stress. Nevertheless, while our primary correlations survived FDR adjustment, they must still be interpreted with caution due to the small sample size, and any associations outside of this defined family remain strictly exploratory and hypothesis-generating. Interestingly, the minimum oxygen saturation (SpO2 nadir) did not demonstrate a statistically significant postoperative improvement (Table 3). This may reflect limited statistical power, measurement variability, or the absence of a true short-term change. Furthermore, when interpreting these respiratory metrics, the methodological limitations of single-lead PPG/CPC relative to gold-standard polysomnography (PSG) must be explicitly considered; as recently highlighted by Murray et al. (2025), home sleep apnea testing devices in pediatric cohorts remain subject to device-related artifacts and require cautious interpretation compared to multi-channel hospital PSG [40].

Considering atrial conduction, although the correlation between Δ P-wave duration and Δ Obstructive event duration was moderate (r = 0.512), the result remained statistically non-significant (*p* = 0.107), likely reflecting the limited sample size of the subgroup analysis.

### 4.4. The Role of Portable Technology

From a translational perspective, this study supports the use of handheld, single-lead ECG devices (KardiaMobile^®^) as a tool to implement for cardiac evaluation in research. While 12-lead ECGs remain the gold standard to fully capture spatial electrical dispersion and phenomena such as QT dispersion, they are often impractical for routine high-frequency research screening in pediatric clinics. Our results suggest that morphometric data from a 30-s Lead I tracing may be sufficient to capture some morphometric ECG changes associated with ATH. This portable cardiac assessment was effectively complemented by SleepImage^®^ technology, which utilizes CPC to provide a portable domiciliary assessment, bypassing the logistical and financial barriers of hospital-based PSG. Furthermore, our choice of a 3-month follow-up period aligns with the American Academy of Otolaryngology—Head and Neck Surgery consensus, allowing for full surgical recovery and the stabilization of sleep architecture [41].

### 4.5. Future Perspectives

These preliminary findings identify P-wave changes and increased QRS amplitude as exploratory correlates of surgical intervention. Furthermore, because ATH induces systemic inflammation and oxidative stress [42,43], future studies combining ECG monitoring with systemic biomarkers and continuous blood pressure evaluation during nighttime sleep stages could provide deeper mechanistic insights. In parallel, future large-scale studies could establish normative thresholds for these ECG shifts and facilitate their integration into postoperative protocols. Such a scalable approach could help to prioritize follow-up PSG for patients with severe preoperative respiratory indices, providing a non-invasive means to distinguish complete recovery from residual disease. While the clinical application of these markers is still nascent, the alignment of portable cardiac and sleep technologies paves the way for a more personalized and accessible approach to pediatric sleep medicine.

### 4.6. Strengths and Limitations

The strengths of this pilot study lie in its prospective, longitudinal design and the application of multivariate analyses to account for demographic confounders. Notably, this research addresses a significant gap in pediatric sleep medicine as the first to characterize QRS morphology changes following AT. However, several limitations must be acknowledged. While the ECG cohort (n = 25) provided significant results, the sleep metric subgroup analysis was constrained by a smaller sample size (n = 11). While we utilized FDR adjustments for our post hoc exploratory correlation family (Δ QRS amplitude max) to mitigate Type I errors, *p*-values for the remaining variables in the broader correlation matrix were not adjusted for multiple comparisons. Consequently, outside of this specific FDR-adjusted family, all other reported correlations must be viewed strictly as exploratory and hypothesis-generating. Furthermore, although Spearman rank tests were utilized as sensitivity analyses, the small sample size limits the overall statistical power. Additionally, our use of a single-lead device (Lead I) primarily reflects the electrical activity of the cardiac lateral wall and does not capture the heart’s full three-dimensional electrical heterogeneity. Consequently, a comprehensive evaluation of all electrocardiographic patterns, including the assessment of QT dispersion—which has been reported to be altered in pediatric OSDB—was impossible in this cohort. Standard 12-lead ECG remains the gold standard for full spatial evaluation. Finally, the three-month follow-up limits longitudinal analysis of ECG pattern. This pilot description aims to describe the method and stimulate interest in performing larger observational studies on this topic.

## 5. Conclusions

AT in children with ATH may induce quantifiable alterations in cardiac electrical parameters. These ECG modifications—specifically P-wave changes and QRS amplitude increase—may represent preliminary ECG changes associated with postoperative respiratory improvement. Single-lead ECG morphometry and nocturnal cardiopulmonary coupling devices represent an attractive approach for future studies. Larger controlled studies are needed to determine whether these ECG parameters can serve as reliable markers of cardiopulmonary recovery.

## Figures and Tables

**Figure 1:**
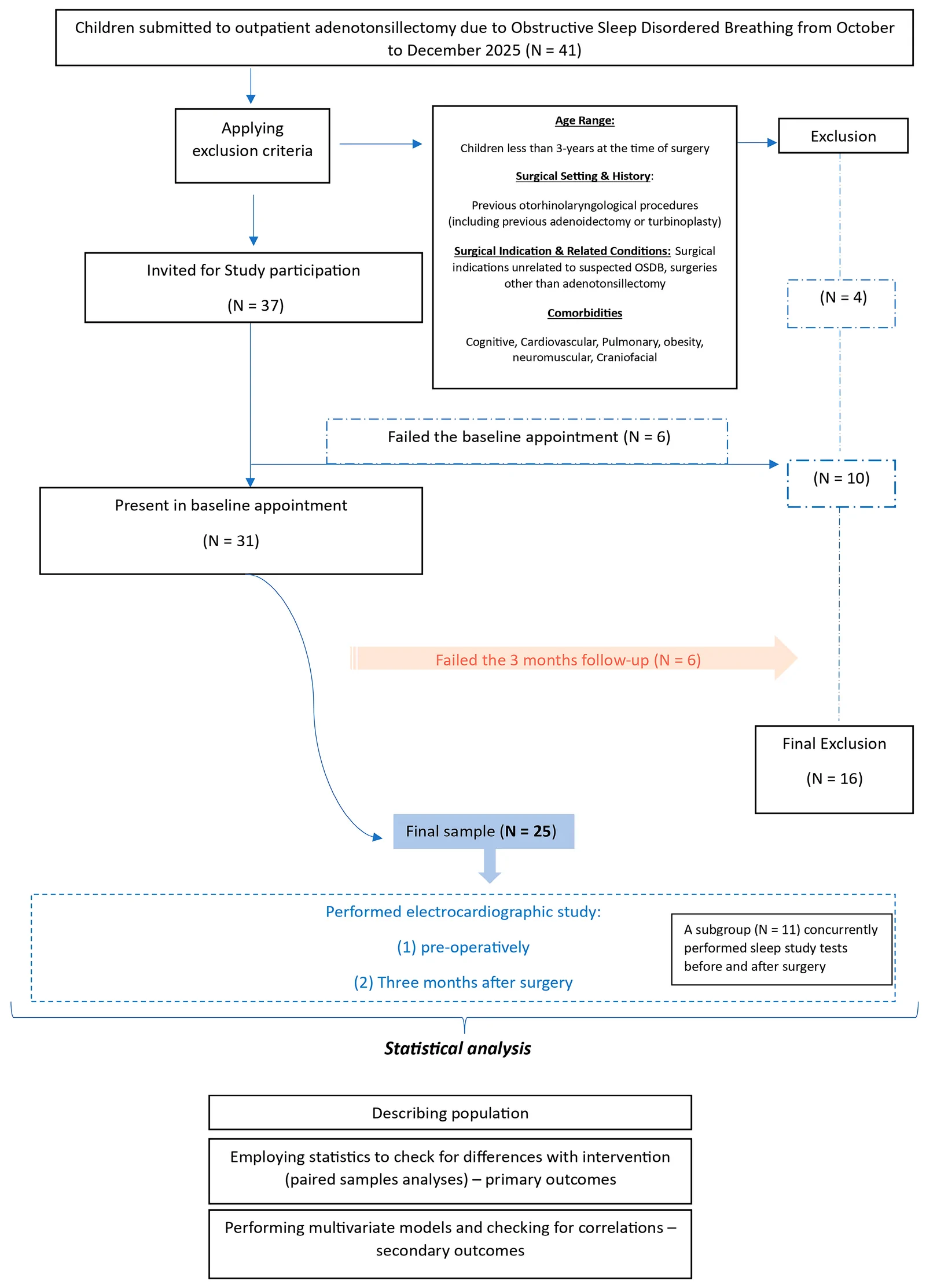
Flowchart of the methodological approach.

**Figure 2:**
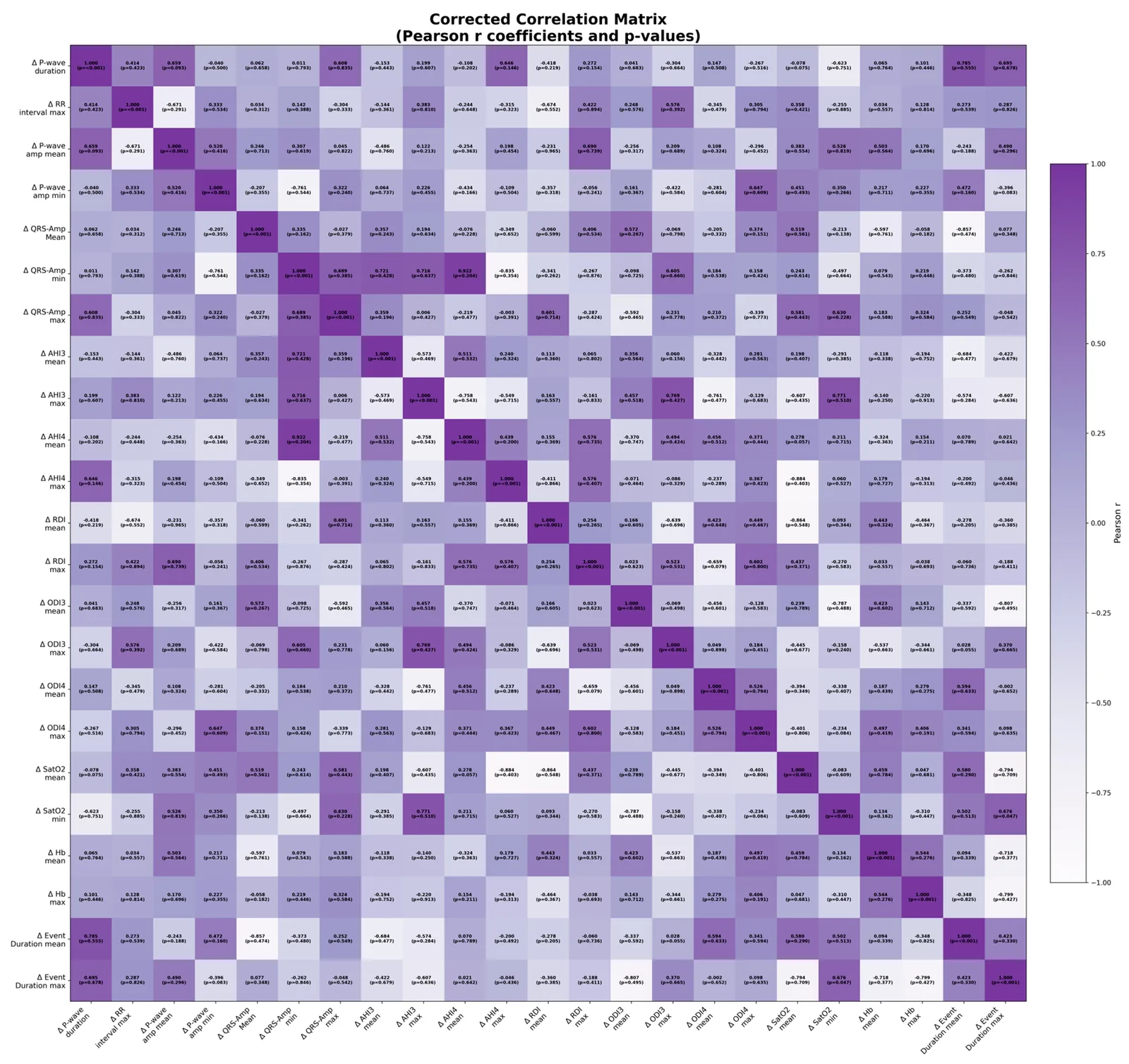
Uncorrected correlation matrix between electrocardiographical and cardiorespiratory significant findings. Δ represents the change in each metric, calculated as the post-intervention value minus the pre-intervention value; Pearson r denotes the correlation coefficient (ranging from −1.0 to 1.0); *p*-value indicates the level of statistical significance, where values < 0.05 are considered significant; RR interval MAX is the maximum R-R interval (the time between heartbeats) reflecting heart rate variability; P-wave dur mean is the mean duration of the P-wave, and P-wave amplitude mean and P-wave amplitude min are the mean and minimum amplitudes of the P-wave, respectively, representing atrial depolarization; QRS-Amp Mean, QRS-Amp min, and QRS-amp Max denote the mean, minimum, and maximum amplitudes of the QRS complex, representing ventricular depolarization; Mean ODI 4%, Max ODI 4%, and Mean ODI 3% refer to the Oxygen Desaturation Indices at the 4% and 3% thresholds, indicating the number of desaturations per hour of sleep; Max AHI 4% signifies the maximum Apnea–Hypopnea Index, or the total number of events per hour; Max HB denotes the maximum hypoxic burden recorded; and Apnea Duration represents the duration of recorded apnea events.

**Table 1:** Comparison of electrocardiographic morphometric and conduction parameters before and three months after adenotonsillectomy (N = 25).

Electrocardiographic Parameter	Variable	Level of Analysis	Pre-Op Mean (SD)	Post-Op Mean (SD)	Delta (Δ)	*p*-Value (2-Tailed)
Conduction velocity/Pace	Heart Rate (beats per minute)	Not applicable	87.84 (10.89)	90.52 (10.07)	+2.68	0.250
	RR interval (milliseconds)	Mean	694.40 (84.66)	670.40 (76.78)	−24.00	0.173
		Min	602.00 (82.76)	610.80 (71.176)	+8.80	0.552
		Max	800.80 (117.82)	737.20 (96.50)	−63.60	**0.027**
P-wave	Duration (milliseconds)	Mean	84.40 (6.51)	81.20 (3.317)	−3.20	**0.043**
		Min	66.00 (7.64)	62.40 (4.359)	−3.60	0.059
		Max	103.20 (6.90)	101.20 (3.317)	−2.00	0.233
	Amplitude (millivolts)	Mean	0.076 (0.008)	0.080 (0.004)	+0.004	**<0.001**
		Min	0.047 (0.005)	0.051(0.006)	+0.004	**0.009**
		Max	0.110 (0.014)	0.116 (0.007)	+0.006	0.054
QRS complex	Duration (milliseconds)	Mean	74.60 (4.77)	76.00 (5.00)	+1.40	0.356
		Min	60.40 (3.51)	60.80 (2.77)	+0.40	0.664
		Max	90.00 (8.66)	90.00 (9.129)	0	1.000
	Amplitude (millivolts)	Mean	0.625 (0.148)	0.724 (0.038)	+0.099	**0.003**
		Min	0.476 (0.079)	0.542(0.116)	+0.066	**0.018**
		Max	0.844 (0.117)	0.902 (0.042)	+0.058	**0.016**

Note: Data are presented as mean (standard deviation). *p*-values were derived from paired *t*-tests. Statistically significant differences (*p* < 0.05) are highlighted in bold; Pre-Op: preoperatively; Post-Op: three months postoperatively.

**Table 2:** Exploratory Linear Mixed Models: adjusted effect of surgery (time) on electrocardiographic parameters conditional on age and sex.

ECG Parameter	Adjusted Effect of Time (β) [95% CI]	SE	*p*-Value
RR Interval (ms)			
Max	−70.9 [−138.3 to −3.6]	34.4	0.039
P-Wave Duration (ms)			
Mean	−4.5 [−8.6 to −0.5]	2.1	0.028
P-Wave Amplitude (mV)			
Mean	+0.007 [0.004 to 0.011]	0.002	<0.001
Min	+0.003 [0.000 to 0.005]	0.001	0.053
Max	+0.012 [0.004 to 0.019]	0.004	0.002
QRS Amplitude (mV)			
Mean	+0.114 [0.064 to 0.163]	0.025	<0.001
Min	+0.105 [0.060 to 0.149]	0.022	<0.001
Max	+0.105 [0.042 to 0.167]	0.032	0.001

Note: The adjusted effect of time (β coefficient) represents the estimated mean change in the electrocardiographic parameter from the preoperative baseline to the three-month postoperative assessment, conditional on the covariates of age and sex. A positive β indicates a postoperative increase in the metric, whereas a negative β indicates a decrease. The models utilized a random intercept for each participant, Restricted Maximum Likelihood (REML) estimation, a variance components covariance structure, and Satterthwaite approximations for degrees of freedom (df = 21). Statistical significance is established at *p* < 0.05. Abbreviations: CI, confidence interval; ms, milliseconds; mV, millivolts; SE, Standard Error.

**Table 3:** Subgroup analysis of 11 patients: paired-samples analysis of peak and mean sleep metrics pre- and postoperatively.

Variable	Level of Analysis	Pre-Op Mean (SD)	Post-Op Mean (SD)	Delta (Δ)	*p*-Value (2-Tailed)
ODI 3%	Peak (Max)	9.63 (14.17)	3.21 (5.21)	−6.42	0.051
	Nightly Mean	3.97 (4.24)	2.59 (5.13)	−1.38	0.043
ODI 4%	Peak (Max)	5.48 (9.36)	1.28 (1.97)	−4.2	0.100
	Nightly Mean	1.95 (2.62)	0.96 (1.89)	−0.99	0.013
Hypoxic burden (% min/h)	Peak (Max)	21.55 (30.98)	11.75 (9.99)	−9.81	0.193
	Nightly Mean	11.10 (10.07)	9.62 (9.39)	−1.48	0.326
SpO2 minimum (nadir) (%)	Peak (Min)	88.00 (4.40)	88.36 (5.16)	+0.36	0.844
	Nightly Mean	91.08 (2.87)	90.35(4.48)	−0.74	0.488
RDI (events/h)	Peak (Max)	21.55 (8.33)	17.26 (8.07)	−4.29	0.098
	Nightly Mean	17.29 (6.31)	14.93 (7.94)	−2.36	0.299
AHI 3% (Global) (events/h)	Peak (Max)	11.61 (6.62)	7.59 (6.63)	−4.02	0.007
	Nightly Mean	8.81 (4.26)	6.40 (6.30)	−2.41	0.112
AHI 4% (Global) (events/h)	Peak (Max)	2.76 (3.26)	0.75 (0.74)	−2.01	0.040
	Nightly Mean	1.20 (0.91)	0.53 (0.68)	−0.67	0.008
Event Duration (seconds)	Peak (Max)	43.45 (8.64)	34.82 (10.76)	−8.63	0.043
	Nightly Mean	19.97 (3.69)	16.48 (0.76)	−3.49	0.009

Data are presented as mean (standard deviation). *p*-values are derived from paired-samples *t*-tests, with statistical significance established at *p* < 0.05. Delta (Δ) values represent means for the calculated longitudinal change (postoperative minus preoperative). Abbreviations: AHI, Apnea–Hypopnea Index (average events per hour); nadir, lowest oxygen saturation recorded; ODI, Oxygen Desaturation Index (desaturation events ≥ 3% or 4%); RDI, Respiratory Disturbance Index; SpO2, peripheral oxygen saturation.

**Table 4:** Post hoc correlation family: Δ QRS amplitude max versus sleep metrics (FDR-adjusted).

Sleep Metric (Δ)	Pearson r [95% CI]	Unadjusted *p*-Value	FDR-Adjusted *p*-Value	Spearman ρ	Spearman *p*-Value
Oxygen Desaturation Index (ODI)					
Δ ODI 3% mean	−0.19 [−0.71 to 0.46]	0.580	0.663	−0.31	0.357
Δ ODI 4% mean	−0.82 [−0.95 to −0.43]	0.002	**0.033**	−0.77	0.006
Δ ODI 3% max	−0.76 [−0.94 to −0.30]	0.006	**0.045**	−0.81	0.002
Δ ODI 4% max	−0.70 [−0.92 to −0.17]	0.016	0.052	−0.73	0.011
Apnea–Hypopnea Index (AHI)					
Δ AHI 3% mean	0.45 [−0.20 to 0.83]	0.161	0.319	0.18	0.588
Δ AHI 4% mean	−0.48 [−0.84 to 0.17]	0.133	0.319	−0.57	0.064
Δ AHI 3% max	0.06 [−0.56 to 0.64]	0.850	0.850	0.05	0.893
Δ AHI 4% max	−0.73 [−0.92 to −0.23]	0.011	**0.045**	−0.62	0.044
Hypoxic Burden					
Δ Hypoxic Burden mean	−0.47 [−0.84 to 0.18]	0.141	0.319	−0.74	0.010
Δ Hypoxic Burden max	−0.73 [−0.92 to −0.23]	0.011	**0.045**	−0.85	0.001
SpO2 Nadir (Minimum)					
Δ SpO2 nadir mean	0.08 [−0.55 to 0.65]	0.819	0.850	0.14	0.686
Δ SpO2 nadir max	0.32 [−0.35 to 0.77]	0.337	0.490	0.49	0.131
Respiratory Disturbance Index (RDI)					
Δ RDI mean	0.41 [−0.26 to 0.81]	0.215	0.344	0.28	0.403
Δ RDI max	0.26 [−0.40 to 0.74]	0.442	0.548	0.19	0.579
Event Duration					
Δ Event Duration mean	−0.44 [−0.82 to 0.22]	0.180	0.319	−0.49	0.124
Δ Event Duration max	−0.26 [−0.74 to 0.41]	0.445	0.548	−0.04	0.904

Note: Δ (Delta) represents the longitudinal change, calculated as the postoperative value minus the preoperative value. This table presents the complete post hoc defined family of 16 comparisons evaluating the relationship between Δ QRS amplitude max and all assessed sleep metrics. Pearson r denotes the linear correlation coefficient alongside its 95% confidence interval (CI). The unadjusted *p*-value reflects the initial significance level, whereas the FDR-adjusted *p*-value indicates significance after applying the Benjamini-Hochberg False Discovery Rate (FDR) correction across the entire family. Spearman ρ (rho) and its associated *p*-value are provided as non-parametric sensitivity analyses to confirm the robustness of the findings against distributional assumptions. Statistical significance is established at *p* < 0.05 (significant FDR-adjusted values are highlighted in bold). Abbreviations: AHI, Apnea–Hypopnea Index; CI, confidence interval; FDR, False Discovery Rate; max, maximum; ODI, Oxygen Desaturation Index; RDI, Respiratory Disturbance Index; SpO2, peripheral oxygen saturation.

## Data Availability

The datasets generated and/or analyzed during the current study are not publicly available due to ethical and privacy restrictions concerning pediatric participant data but may be available from the corresponding author upon reasonable request.

## Abbreviations

The following abbreviations are used in this manuscript:

AHI	Apnea–Hypopnea Index
AT	Adenotonsillectomy
ATH	Adenotonsillar Hypertrophy
Bpm	Beats Per Minute
CHAT	Childhood Adenotonsillectomy Trial
CMIN	Centro Materno-Infantil do Norte
CPC	Cardiopulmonary Coupling
ECG	Electrocardiogram/Electrocardiographic
FDA	Food and Drug Administration
HRV	Heart Rate Variability
ICBAS	Instituto de Ciências Biomédicas Abel Salazar
LMMs	Linear Mixed Models
MPI	Myocardial Performance Index
Ms	Milliseconds
mV	Millivolts
NREM	Non-Rapid Eye Movement
ODI	Oxygen Desaturation Index
OSA	Obstructive Sleep Apnea
OSDB	Obstructive Sleep-Disordered Breathing
PDR	Pulse-Derived Respiration
PPG	Photoplethysmogram
PWD	P-wave Dispersion
RA	Right Atrial
RDI	Respiratory Disturbance Index
REM	Rapid Eye Movement
RV	Right Ventricular
sAHI	Stage-specific Apnea–Hypopnea Index
SD	Standard Deviation
SpO2	Peripheral Oxygen Saturation
STROBE	Strengthening the Reporting of Observational Studies in Epidemiology
TDI	Tissue Doppler Imaging
ULSSA	Unidade Local de Saúde de Santo António

## References

[1] Mitchell R.B., Archer S.M., Ishman S.L., Rosenfeld R.M., Coles S., Finestone S.A., Friedman N.R., Giordano T., Hildrew D.M., Kim T.W. (2019). Clinical Practice Guideline: Tonsillectomy in Children (Update)-Executive Summary. Otolaryngol. Neck Surg. Off. J. Am. Acad. Otolaryngol. Neck Surg..

[2] Redline S., Cook K., Chervin R.D., Ishman S., Baldassari C.M., Mitchell R.B., Tapia I.E., Amin R., Hassan F., Ibrahim S. (2023). Adenotonsillectomy for Snoring and Mild Sleep Apnea in Children: A Randomized Clinical Trial. JAMA.

[3] Deshpande P., Salcedo B., Haq C. (2022). Common Sleep Disorders in Children. Am. Fam. Physician.

[4] Blum R.H., McGowan F.X.J. (2004). Chronic upper airway obstruction and cardiac dysfunction: Anatomy, pathophysiology and anesthetic implications. Paediatr. Anaesth..

[5] Mediano O., López-Monzoni S., Castillo-García M., Romero-Peralta S., Viejo-Ayuso M.E., Silgado-Martínez L., Álvarez-Balado L., Forné C., Resano-Barrio P., García-Rio F. (2025). Cardiovascular Risk Through Hypoxic Burden in Children With Sleep Apnea: A Secondary Analysis of a Nonrandomized Clinical Trial. JAMA Netw. Open.

[6] Patino M., Sadhasivam S., Mahmoud M. (2013). Obstructive sleep apnoea in children: Perioperative considerations. Br. J. Anaesth..

[7] Thomas S., Patel S., Gummalla P., Tablizo M.A., Kier C. (2022). You Cannot Hit Snooze on OSA: Sequelae of Pediatric Obstructive Sleep Apnea. Children.

[8] Javaheri S., Javaheri S., Somers V.K., Gozal D., Mokhlesi B., Mehra R., McNicholas W.T., Zee P.C., Campos-Rodriguez F., Martinez-Garcia M.A. (2024). Interactions of Obstructive Sleep Apnea with the Pathophysiology of Cardiovascular Disease, Part 1: JACC State-of-the-Art Review. J. Am. Coll. Cardiol..

[9] Baker-Smith C.M., Isaiah A., Melendres M.C., Mahgerefteh J., Lasso-Pirot A., Mayo S., Gooding H., Zachariah J. (2021). Sleep-Disordered Breathing and Cardiovascular Disease in Children and Adolescents: A Scientific Statement from the American Heart Association. J. Am. Heart Assoc..

[10] Kong F., Sun Y.-L., Yuan B. (2022). Prevalence of pulmonary hypertension in children with adenoid or adenotonsillar hypertrophy: A meta-analysis. Int. J. Pediatr. Otorhinolaryngol..

[11] Smith D.F., Amin R.S. (2019). OSA and Cardiovascular Risk in Pediatrics. Chest.

[12] Kocabaş A., Salman N., Ekici F., Cetin I., Akcan F.A. (2014). Evaluation of cardiac functions and atrial electromechanical delay in children with adenotonsillar hypertrophy. Pediatr. Cardiol..

[13] Sameema V.V., Soni K., Deora S., Sharma J.B., Choudhury B., Kaushal D., Chhabra S., Goyal A. (2022). Assessment of preoperative and postoperative cardiac function in children with adenotonsillar hypertrophy: A prospective cohort study. Eur. Arch. Oto-Rhino-Laryngol..

[14] Ozbilgic T., Suslu A.E., Aykan H.H., Pehlivanoglu B., Onal D., Kasikci M., Duzova A., Emiralioglu N., Yalcin E.E., Ersoz D.D. (2022). The impact of the Adenotonsillectomy on cardiac functions and oxidative stress. Int. J. Pediatr. Otorhinolaryngol..

[15] Lemyze M., Mallat J. (2014). Understanding negative pressure pulmonary edema. Intensive Care Med..

[16] Domany K.A., Hossain M.M., Nava-Guerra L., Khoo M.C., McConnell K., Carroll J.L., Xu Y., DiFrancesco M., Amin R.S. (2018). Cardioventilatory control in preterm-born children and the risk of obstructive sleep apnea. Am. J. Respir. Crit. Care Med..

[17] Domany K.A., Huang G., Hossain M.M., Schuler C.L., Somers V.K., Daniels S.R., Amin R. (2021). Effect of Adenotonsillectomy on Cardiac Function in Children Age 5–13 Years with Obstructive Sleep Apnea. Am. J. Cardiol..

[18] Yeghiazarians Y., Jneid H., Tietjens J.R., Redline S., Brown D.L., El-Sherif N., Mehra R., Bozkurt B., Ndumele C.E., Somers V.K. (2021). Obstructive Sleep Apnea and Cardiovascular Disease: A Scientific Statement from the American Heart Association. Circulation.

[19] Mehra R., Chung M.K., Olshansky B., Dobrev D., Jackson C.L., Kundel V., Linz D., Redeker N.S., Redline S., Sanders P. (2022). Sleep-Disordered Breathing and Cardiac Arrhythmias in Adults: Mechanistic Insights and Clinical Implications: A Scientific Statement from the American Heart Association. Circulation.

[20] Cowie M.R., Linz D., Redline S., Somers V.K., Simonds A.K. (2021). Sleep Disordered Breathing and Cardiovascular Disease: JACC State-of-the-Art Review. J. Am. Coll. Cardiol..

[21] Goette A., Kalman J.M., Aguinaga L., Akar J., Cabrera J.A., Chen S.A., Chugh S.S., Corradi D., D’Avila A., Dobrev D. (2016). EHRA/HRS/APHRS/SOLAECE expert consensus on Atrial cardiomyopathies: Definition, characterisation, and clinical implication. J. Arrhythmia.

[22] Attia G., Ahmad M.A., Saleh A.B., Elsharkawy A. (2010). Impact of obstructive sleep apnea on global myocardial performance in children assessed by tissue Doppler imaging. Pediatr. Cardiol..

[23] Solano-Pérez E., Coso C., Romero-Peralta S., Castillo-García M., López-Monzoni S., Ortigado A., Mediano O. (2024). New Approaches to the Management of Cardiovascular Risk Associated with Sleep Respiratory Disorders in Pediatric Patients. Biomedicines.

[24] Sun Y.-L., Yuan B., Kong F., Li X.-M. (2022). Effects of adenoidectomy or adenotonsillectomy on the cardiovascular system in children: A meta-analysis. Eur. Arch. Oto-Rhino-Laryngol..

[25] Kelmanson I.A. (2023). Increased P-wave dispersion in patients with obstructive sleep apnea syndrome: A meta-analysis. Sleep Breath.

[26] Kraikriangsri C., Khositseth A., Kuptanon T. (2019). P-wave dispersion as a simple tool for screening childhood obstructive sleep apnea syndrome. Sleep Med..

[27] Hilmisson H., Berman S., Magnusdottir S. (2020). Sleep apnea diagnosis in children using software-generated apnea-hypopnea index (AHI) derived from data recorded with a single photoplethysmogram sensor (PPG). Sleep Breath.

[28] Hilmisson H., Lange N., Magnusdottir S. (2019). Objective sleep quality and metabolic risk in healthy weight children results from the randomized Childhood Adenotonsillectomy Trial (CHAT). Sleep Breath.

[29] de Sousa F.A., Rios M., Branco P., Azevedo S.R., Santos M., de Magalhães M.F. (2025). Paediatric Obstructive Sleep Disordered Breathing: Cardiovascular Changes After Adenotonsillectomy. Clin. Otolaryngol..

[30] Bhattacharjee R., Kim J., Kheirandish-Gozal L., Gozal D. (2011). Obesity and obstructive sleep apnea syndrome in children: A tale of inflammatory cascades. Pediatr. Pulmonol..

[31] Varela M., Roy A., Lee J. (2021). A survey of pathways for mechano-electric coupling in the atria. Prog. Biophys. Mol. Biol..

[32] Medvedev R.Y., Afolabi S.O., Turner D.G.P., Glukhov A.V. (2024). Mechanisms of stretch-induced electro-anatomical remodeling and atrial arrhythmogenesis. J. Mol. Cell. Cardiol..

[33] Hancock E.W., Deal B.J., Mirvis D.M., Okin P., Kligfield P., Gettes L.S. (2009). AHA/ACCF/HRS Recommendations for the Standardization and Interpretation of the Electrocardiogram. Circulation.

[34] Chen Y.C., Voskoboinik A., La Gerche A., Marwick T.H., McMullen J.R. (2021). Prevention of Pathological Atrial Remodeling and Atrial Fibrillation: JACC State-of-the-Art Review. J. Am. Coll. Cardiol..

[35] Joglar J.A., Chung M.K., Armbruster A.L., Benjamin E.J., Chyou J.Y., Cronin E.M., Deswal A., Eckhardt L.L., Goldberger Z.D., Gopinathannair R. (2024). 2023 ACC/AHA/ACCP/HRS Guideline for the Diagnosis and Management of Atrial Fibrillation: A Report of the American College of Cardiology/American Heart Association Joint Committee on Clinical Practice Guidelines. Circulation.

[36] de Sousa F.A., Pinho M.R., Pinto A.N., Coutinho M.B., Afonso A.C., Magalhães M.F. (2024). Modelling metabolic performance in paediatric obstructive sleep disordered breathing: A case–control study. J. Sleep Res..

[37] Bacharova L., Triantafyllou E., Vazaios C., Tomeckova I., Paranicova I., Tkacova R. (2015). The effect of obstructive sleep apnea on QRS complex morphology. J. Electrocardiol..

[38] Marcus C.L., Moore R.H., Rosen C.L., Giordani B., Garetz S.L., Taylor H.G., Mitchell R.B., Amin R., Katz E.S., Arens R. (2013). A randomized trial of adenotonsillectomy for childhood sleep apnea. N. Engl. J. Med..

[39] Isaiah A., Bertoni D., Pereira K.D., Diaz-Abad M., Mitchell R.B., Das G. (2020). Treatment-Related Changes in Heart Rate Variability in Children with Sleep Apnea. Otolaryngol. Neck Surg..

[40] Murray A.M., Matarese C.A., Lloyd R.M., Sorensen C., LeMahieu A., Reckward D., Tullis R., Bellinger M., Brown V., Herold D.L. (2026). A photoplethysmography-based home sleep apnea test compared with polysomnography in children 2 to 6 years of age. J. Clin. Sleep Med..

[41] Ishman S.L., Maturo S., Schwartz S., McKenna M., Baldassari C.M., Bergeron M., Chernobilsky B., Ehsan Z., Gagnon L., Liu Y.-C.C. (2023). Expert Consensus Statement: Management of Pediatric Persistent Obstructive Sleep Apnea After Adenotonsillectomy. Otolaryngol. Neck Surg..

[42] Tiboc-Schnell C.N., Filip G.A., Bolboaca S.D., Decea N., Panta P.C., Iacob D., Mihut G., Marin A., Man S.C. (2021). Biomarkers of pediatric obstructive sleep apnea syndrome and the assessment of quality of life before and after adenotonsillectomy. J. Physiol. Pharmacol..

[43] Yucel K., Aydin I., Erdem S.S. (2021). Hypoxia induced factor-1α levels in patients undergoing adenoidectomy. Scand. J. Clin. Lab. Investig..

